# Evaluating confidence in translational science principles to guide workforce development

**DOI:** 10.1017/cts.2026.10772

**Published:** 2026-06-15

**Authors:** Darcy A. Freedman, Holly E. Hartman, Emily S. Nelson, Ann Pearman

**Affiliations:** 1 Case Western Reserve University School of Medicine, Cleveland, OH, USA; 2 MetroHealth Medical Center, Cleveland, OH, USA

**Keywords:** Translational science, capacity building, factor analysis, program evaluation, workforce development

## Abstract

**Background:**

Translational science (TS) workforce development initiatives are emerging to fast-track conversion of findings into applications to improve health. Principles of TS were developed to guide workforce development; however, few tools are available to evaluate confidence related to these principles to benchmark progress.

**Objective:**

Our goal was to develop and validate a scale for assessing confidence related to the seven TS principles and get feedback about future workforce development opportunities that could be evaluated with this scale.

**Methods:**

A cross-sectional survey was conducted in 2025 engaging 158 researchers and research staff affiliated with three Clinical and Translational Science Award hubs in Alabama, Massachusetts, and Ohio. Factor analysis was conducted to determine interrelatedness among the TS principles, reliability was assessed using Cronbach’s alpha, and mixed effects models were used to examine differences in confidence levels.

**Results:**

Four factors emerged (α = 0.95 for full scale, 14 items), including Taking Risks for Impact (α = 0.85, 2 items), Multi-perspective Research (α = 0.88, 3 items), Collaborative Methods (α = 0.89, 6 items), and Efficiency and Productive Failure (α = 0.88, 3 items). Confidence levels varied significantly by factor (*p* < 0.001) with the lowest mean confidence (1 = lowest, 5 = highest) for the Efficiency and Productive Failure (2.56) and Taking Risks for Impact (2.79) factors. There were no differences in mean levels of confidence by demographics, site, or experience.

**Conclusion:**

Findings provide guidance for prioritizing topics for future TS workforce development initiatives as well as a brief scale for evaluating how these interventions cultivate confidence across the seven TS principles.

## Introduction

Translational science (TS) as a field emerged in the early 2000s to reduce barriers to getting research findings applied within real-world settings [1]. It is a field focused on generating scientific and operational solutions to overcome long-standing challenges that limit progress of research along the translational spectrum, from basic science to implementation in clinical and public health practice [2,3].

There is growing interest in TS since less than one percent of all discoveries related to human health reach the real world. When discoveries are translated into evidence-based practices, this process often takes up to 20 years [2]. In some cases, these “solutions” contribute to health disparities due to lack of engagement of all populations in the research process and limited guidance for addressing implementation complexities in different settings, including those with the most unmet needs [4–6]. The gap between understanding a disease and translating these insights into evidence-based solutions for real-world implementation has been described as the “zone of chaos” that is “irreducibly complex” and “immutably unpredictable” [7].

Seven TS principles were developed to guide scientific and operational processes intended to accelerate the movement of research into practice [8,9]. There was an eighth TS principle focused on “diversity, equity, inclusion, and accessibility” [10] that is no longer included [8]. These principles broaden the scope of TS by providing guidance to optimize the conduct of research, such as clinical trials, dissemination and implementation science, and drug development, while streamlining the scientific, operational, financial, regulatory, and administrative capacities that support translational research [9]. The TS principles, considered to be a work in progress, are currently focused on capacities and infrastructure to foster clinical and translational research that (a) prioritizes initiatives focused on unmet needs, (b) yields generalizable solutions for common and persistent problems, (c) emphasize creativity and innovation, (d) leverage cross-disciplinary team science, (e) enhance efficiencies and the speed of research, (f) use boundary-cross partnerships, and (g) apply bold and rigorous strategies [8]. The TS principles complement other resources intended to expand TS workforce capacity, such as TS competencies [9] and TS characteristics [3].

There has been some effort to operationalize the TS principles to support measurement and evaluation. A prior study by Schneider and colleagues converted the TS principles into 24 items, which were applied using content analysis to evaluate the extent to which pilot grant proposals from NCATS-funded Clinical and Translational Science Award (CTSA) hubs were congruent with the principles [11]. Factor analysis conducted using this assessment revealed the TS principles organized into three factors [11].

Building on these developments, our goal here was to create an approach for self-assessment of confidence in the TS principles. We conducted this research with a sample of current TS researchers, research staff, and community partners affiliated with CTSA hubs. Our primary aim was to develop a scale for self-assessing confidence in the TS principles that could be used to evaluate future TS workforce development initiatives. We hypothesized the three factors identified by Scheider and colleagues would also be found in our sample. Our secondary goal was to get feedback for developing future TS workforce development programming, which may be evaluated using the scale we were validating.

## Methods

A cross-sectional survey was conducted from March to May 2025. Data analysis was conducted from June to October 2025. The study was approved and determined to be exempt by the Institutional Review Board at Case Western Reserve University (STUDY20241496).

### Participants

Survey participants were recruited from three CTSA hubs located in Alabama, Massachusetts, and Ohio. Key contacts from each hub shared information about the study with their respective listservs, which included faculty, clinicians, students, trainees, staff, and community partners. Participants were eligible to join the study if they were at least 18 years of age, affiliated with one of the CTSA hubs, and had professional roles and responsibilities that included at least some level of health-related research. Those interested could voluntarily complete an electronic consent form and the survey using REDCap, a secure web-based application for managing online surveys and databases [12,13]. In total, 220 people consented to join the study and 158 (71.8%) completed at least part of the survey. Three surveys were excluded from factor analysis because they indicated none of their time was spent doing research, an inclusion criterion for this analysis.

### Data collection

The online survey included questions about demographics, TS training desires (i.e., preferred length or format of training), and a matrix of 26 TS capacities aligned with the seven TS principles [8], for which participants ranked their confidence on a five-point scale (1 = not at all confident, 2 = slightly confident, 3 = moderately confident, 4 = confident, 5 = very confident). The 26 items were derived from two sources. First, we included items from an existing 24-item assessment used to conduct content analysis of 26 CTSA pilot grant applications based on the TS principles [11]. We included 21 of the 24 items with slight modifications to make the statements more understandable for a self-assessment of confidence versus content analysis of a grant application [11]. For instance, the original item “This project uses a multidisciplinary approach” (yes/no) was changed to “How confident are you in doing research that uses a multidisciplinary approach” (rating of confidence). Second, we added five items that provided concrete examples of the TS principles [8], such as “How confident are you in designing studies that build in processes that reward learning from productive failure?” Participants were asked to think about the following definition of TS when answering these questions: “Translational science comes up with innovations for getting research applied in the real world. It is focused on how to do the science more creatively, how to organize the work and research teams, how to get resources to do research, and/or how to run research processes more effectively and equitably. Translational science solutions can change how research is conducted, making it faster, more relevant, and more impactful.”

### Data analysis

Descriptive statistics were calculated using R version 4.5.1 to summarize data. Factor analysis was conducted using the psych package in R to examine how the 26 TS confidence ratings could be understood as being interrelated and organized into factors [14]. Appropriateness of using factor analysis was determined by examining Pearson’s correlations, the Kaiser–Meyer–Olkin (KMO) index, and Bartlett’s test of sphericity. To determine the number of factors, multiple methods were employed. These methods included eigenvalues of the correlation matrix greater than one (Kaiser’s rule), Cattell’s scree test, and Horn’s parallel analysis. The number of factors in the final model was selected based on model fit including BIC, root mean squared error of approximation (RMSEA), and the Tucker–Lewis Index (TLI). Factor analysis was conducted using oblimin rotation and maximum likelihood. Questions were assigned to a factor based on having a factor loading >0.4. Four items did not meet the factor loading threshold. Cronbach’s alpha was used to gauge internal consistency.

A mixed effect model was used to examine confidence for each item with fixed effects for each factor, a random effect for each individual nested within CTSA hub site, and a random effect for each question. We tested if factors had differing levels of confidence using a likelihood ratio test by running the full and reduced model with maximum likelihood estimators. Subset analyses were done for age group, area of translational research, and role. Models for subset analyses were completed with fewer or no random effects for computational purposes. The lmer4 and lmerTest packages in R were used for the mixed effect analyses.

Iterative thematic qualitative analysis was conducted on two open-ended survey questions. Responses were grouped first based on the general topic of the comment, then consolidated into three parent themes.

## Results

### Survey

As shown in Table 1, participants were mostly White (68%) women (75%) who identified as research staff (35%) or university faculty (33%). Participants represented the spectrum of translational research areas with pre-clinical research (50%) and public health research (44%) being the most common. More than half reported minimal or no prior formal training in TS.

**Table 1.** Characteristics of study participants (*N* = 158) Table 1 long description.

Race	*N*	%
Asian	21	13
Black or African American	16	10
Middle Eastern or Arab American	4	3
White	107	68
American Indian, Alaska Native, Native Hawaiian, Other Pacific Islander, or Multiple Races^a^	5	3
Prefer not to answer	5	3
**Ethnicity,** Hispanic or Latino/a/e	3	2
**Gender,** women	119	75
**Age group**		
18–29 years	21	13
30–45 years	74	47
46–60 years	36	23
60+ years	26	16
Prefer not to answer	1	1
**Professional affiliation, primary sector**		
Healthcare, pharmaceutical, or device	78	49
Education	42	27
Public health	21	13
Community resident, leader, or community-based organization	6	4
Government or business^a^	6	4
**Role**		
Research staff	55	35
University faculty	52	33
Healthcare provider	16	10
Research trainees	16	10
Community partner	10	6
Other	9	6
**Amount of time spent conducting health-related research**		
None (0%)	3	2
Minimal (up to 25%)	39	25
Some (up to 50%)	34	22
A lot (up to 75%)	29	18
Almost all (more than 75%)	52	33
Prefer not to answer	1	1
**Translational research focus^b^**		
Basic research	23	15
Pre-clinical research	79	50
Clinical research	17	11
Implementation research	54	34
Public Health research	69	44
**Prior formal training in translational science**		
None	20	13
Minimal	60	38
Some	53	34
A lot	21	13
Prefer not to answer	4	3

a Multiple items with low numbers combined to protect privacy.

b Participants could select the two that best represents their research.

Twenty-two of the 26 items were incorporated in factor analysis, including one item that loaded on two factors (i.e., doing research that uses a multidisciplinary approach). Average correlation between items was 0.55 (range: 0.20–0.80). The KMO measure of sampling adequacy (MSA) was 0.94, which is “marvelous.” The Barlett’s test was significant indicating the correlation matrix is not an identity matrix. All tests indicated that factor analysis is appropriate for the data. The number of factors was suggested to be two based on the eigenvalues of the correlation matrix, three based on Cattell’s scree test, and four based on Horn’s parallel analysis. Models with 2–5 factors were compared, and we selected the model with four factors based on model fit. The Cronbach’s alpha was 0.95 for the 22 items, including one item that loaded on two factors resulting in 21 unique items, and 0.94 for the brief 14-item scale (described next) indicating excellent internal consistency for the overall scale.

We defined the emergent factors as Taking Risks for Impact (Factor 1) with two items (*α* = 0.85), Multi-perspective Research (Factor 2) with five items (*α* = 0.89), Collaborative Methods (Factor 3) with 10 items (*α* = 0.91), and Efficiency and Productive Failure (Factor 4) with six items (*α* = 0.91) (Table 2). The internal consistencies of the four different factors, including all items, was good to excellent. We assessed the impact of removing items within factors to simplify the scale and found that internal consistencies were maintained when removing eight items within Factors 2, 3, and 4, which reduced the total items from 22 to 14 in the brief scale. The overall and brief scales retained high internal consistency as indicated by changes in Cronbach’s alphas from 0.89 (overall) to 0.88 (brief) for “Multi-perspective Research” (Factor 2), 0.91 (overall) to 0.89 (brief) for “Collaborative Methods” (Factor 3), and from 0.91 (overall) to 0.88 (brief) for “Efficiency and Productive Failure” (Factor 4). Factors 2, 3, and 4 included items from different TS principles indicating interrelatedness among the principles.

**Table 2.** Factor loadings and description of items (*N* = 155). Standardized Cronbach’s alphas are presented

Factors and items related the principles of translational science		Mean score	Factor loading	TS principle	Included in brief scale
Factor 1: Taking Risks for Impact, std alpha = 0.85 (all items)					
1.1	Doing research that encourages transformative ideas and risk taking to increase the chance that research findings will lead to real-world application.	2.88	0.52	Bold & Rigorous^a^	Yes
1.2	Doing research that takes risks through bold, paradigm-challenging, and/or disruptive ideas in your field of study.	2.65	0.489	Bold & Rigorous^a^	Yes
Factor 2: Multi-perspective Research, std alpha = 0.89 (all items) and 0.88 (brief scale)					
2.1	Developing a research project that addresses an unmet scientific, patient, or population health need.	3.63	0.862	Unmet Needs^b^	Yes
2.2	Conducting research leading to knowledge that will be generalizable to a variety of diseases or conditions.	3.50	0.846	Generalizable Solutions^c^	Yes
2.3	Doing research that is innovative in terms of the scientific approach, methods, or processes.	3.41	0.701	Creativity & Innovation^d^	Yes
2.4^h^	Doing research that uses a multidisciplinary approach.	3.77	0.52	Team Science^e^	No
2.5	Doing research that integrates knowledge to develop more holistic findings relevant for real-world application.	3.21	0.465	Team Science^e^	No
Factor 3: Collaboration methods, std alpha = 0.91 (all items) and 0.89 (brief scale)					
3.1	Doing research that includes innovations related to teamwork, partnerships, and/or operations to enhance quality and impact.	3.20	0.777	Creativity & Innovation^d^	Yes
3.2	Incentivizing collaboration through recognition and reward systems that value team science, cross- disciplinary collaboration, patient and community engagement, and cross- sector partnerships.	2.91	0.75	Boundary Spanning^f^	Yes
3.3	Engaging all relevant expertise across disciplines, fields, and/or professions to produce research that advances translation to the real world.	3.21	0.723	Team Science^e^	Yes
3.4^i^	Using methods that support and facilitate meaningful and trustworthy collaboration, community engagement, and partnerships to achieve scientific goals.	3.17	0.698	Boundary Spanning^f^	Yes
3.5	Doing research that will be impactful for real-world application.	3.70	0.661	Bold & Rigorous^a^	Yes
3.6	Implementing evidence-based practices to enhance the speed at which research teams develop shared goals and improve team communication and coordination of work tasks to achieve these goals.	3.04	0.568	Efficiency & Speed^g^	Yes
3.7	Implementing evidence-informed practices for collaborations, engagement, and partnership to support research.	3.58	0.542	Boundary Spanning^f^	No
3.8	Doing research that has the goal of making research processes more efficient or effective.	3.15	0.496	Efficiency & Speed^g^	No
3.9	Doing research that is likely to transform health outcomes in the real world.	3.23	0.467	Bold & Rigorous^a^	Yes
3.10^h^	Doing research that uses a multidisciplinary approach.	3.77	0.44	Team Science^e^	No
Factor 4: Efficiency and Productive Failure, std alpha = 0.91 (all items) and 0.88 (brief scale)					
4.1	Implementing rewards for efficiency in research by encouraging rapid failures and redirection of resources to subsequent attempts.	2.15	0.896	Efficiency & Speed^g^	Yes
4.2^i^	Designing studies that build in processes that reward learning from productive failures.	2.48	0.707	Bold & Rigorous^a^	Yes
4.3^i^	Doing research with the goal of fundamentally changing the translational science ecosystem.	2.33	0.636	Bold & Rigorous^a^	Yes
4.4	Implementing milestone-based decision making in research to enable rapid agreement on go/no-go decisions to enable resources to be used most efficiently.	2.54	0.621	Efficiency & Speed^g^	No
4.5	Doing research that tries to address common roadblocks or bottlenecks in translational research.	2.8	0.58	Generalizable Solutions^c^	No
4.6	Doing research focused on developing and implementing innovations in scientific approaches, methods, and/or technologies to accelerate the pace of translational research.	2.83	0.399	Efficiency & Speed^g^	No
**Items not included in any factor due to loadings below 0.40.**					
A	Doing research that approaches challenges and the development of solutions by learning from commonalities across research on a range of diseases and conditions.	2.91	<0.40	Generalizable Solutions^c^	–
B	Doing research that integrates concepts, theories, methods, technologies, and/or approaches from the range of disciplines, fields, and professions that can contribute to advancing your research goals.	3.15	<0.40	Team Science^e^	–
C^i^	Proposing research that is focused on historically intractable problems or research with a high risk of failure.	2.82	<0.40	Bold & Rigorous^a^	–
D^i^	Providing leadership, policies, and physical spaces that facilitate boundary-crossing research collaborations within and outside of your organization.	2.64	<0.40	Boundary Spanning^f^	–

TS = translational science.

a Use bold and rigorous research approaches.

b Prioritize initiatives that address unmet needs.

c Generalizable solutions.

d Emphasize creativity and innovation.

e Leverage cross-disciplinary team science.

f Utilize boundary-spanning partnerships to advance translation.

g Enhancing efficiency and speed in translational research.

h Item loaded on two factors.

i Item developed by research team in alignment with TS principles.

**Figure 1.**
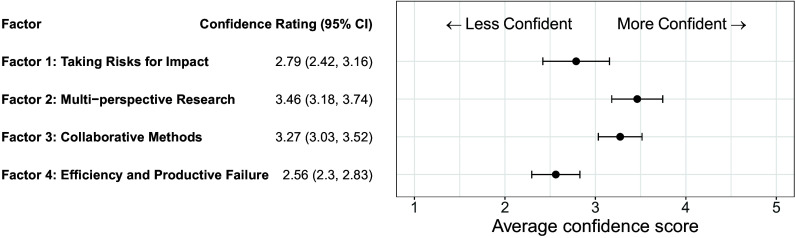
Confidence in translational science principles organized by factors. Plot depicting level of self-reported confidence by the four translational science factors (*N* = 155). Mean confidence scores and 95% confidence intervals are included. The four factors (mean scores) include Factor 1: Taking Risks for Impact (2.79), Factor 2: Multi-perspective Research (3.46), Factor 3: Collaborative Methods (3.27), and Factor 4: Efficiency and Productive Failure (2.56).

**Figure 2.**
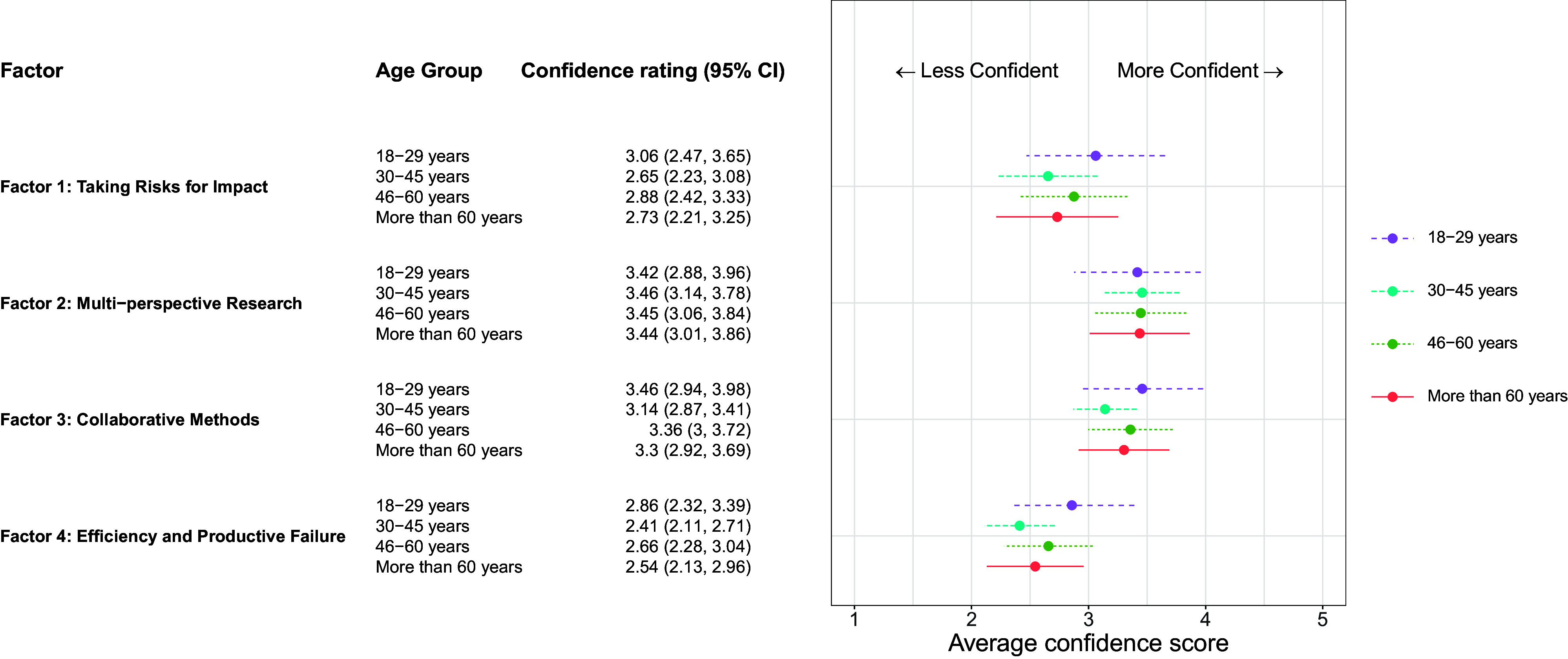
Figure 2 long description. Confidence in translational science principles organized by participant age. Plot depicting level of self-reported confidence by the four translational science factors among participants by age group (*N* = 155). Mean confidence scores and 95% confidence intervals are included. The four age groups are based on self-report including 18–29 years (purple), 30–45 years (turquoise), 46–60 years (green), and more than 60 years (red). There were no significant differences by age group across the four factors.

**Figure 3.**
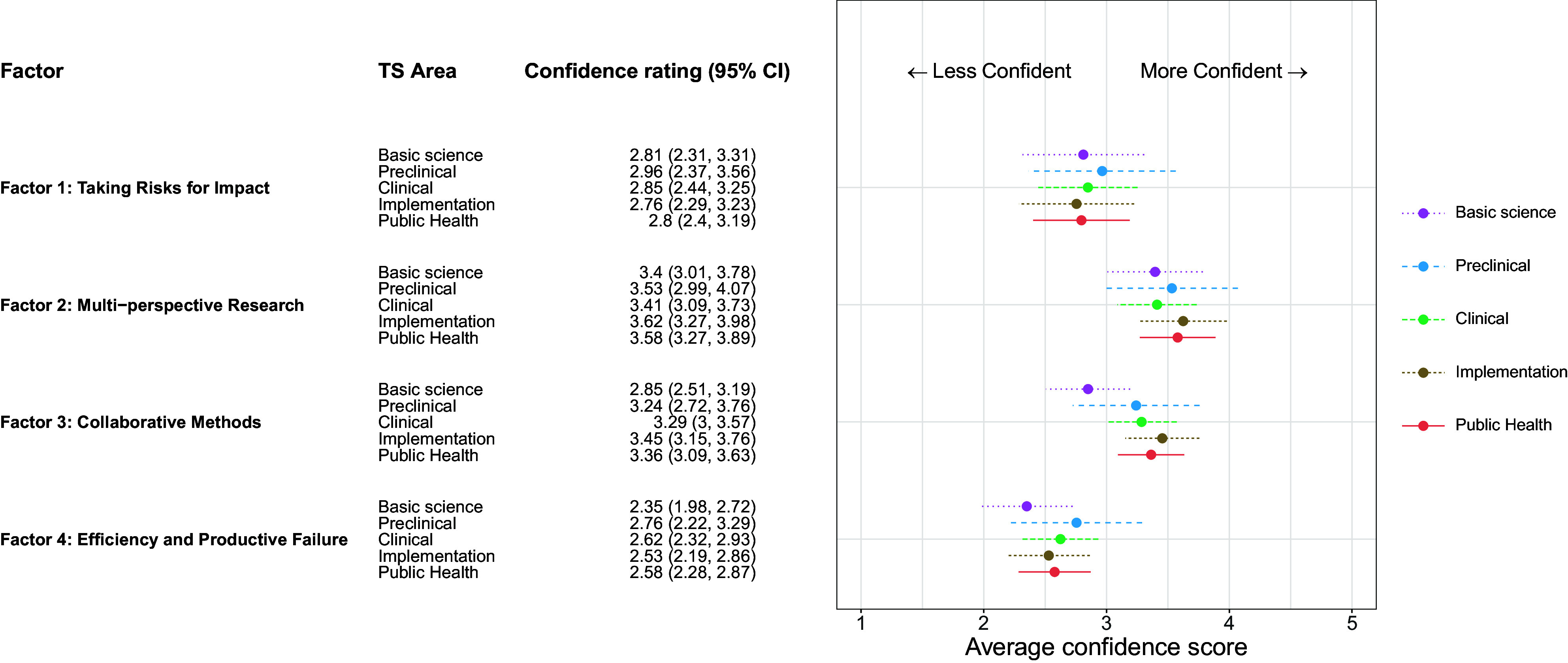
Confidence in translational science principles organized by type of translational research. Plot depicting level of self-reported confidence by the four translational science factors among participants by type of translational research (*N* = 155). Mean confidence scores and 95% confidence intervals are included. The five types of translational research are based on self-report including basic science (pink), pre-clinical (blue), clinical (green), implementation (brown), and public health (red). There were no significant differences by type of translational research across the four factors.

Among participants from the three CTSA hubs, confidence on all four factors was in the moderate range (Figure 1). Confidence levels varied significantly by factor (*p* < 0.001). Confidence was lowest for the factor pertaining to Efficiency and Productive Failure (Factor 4) with the mean score of 2.56 (95% Confidence Interval: 2.3-2.83) followed by the factor pertaining to Taking Risks for Impact (Factor 1) with a mean score of 2.79 (95% Confidence Interval: 2.42–3.16). These trends did not change based on subgroup analyses comparing different age groups (Figure 2), people focused on different areas of translational research (Figure 3), and different roles of participants.

Lastly, we examined preferences for training related to TS. In terms of format, most participants preferred online synchronous (60%) or online asynchronous sessions (57%). In terms of length, 71% reported sessions should be one to 1.5 hours each for a median of 17 hours of TS training time over one year (approximately 1.5 hours per month). For training content, respondents prioritized gaining access to tools and resources to use after the training, learning about best practices for TS, and getting hands-on experience applying TS topics during the training. We asked participants for their open-ended advice for developing future TS trainings, and the top themes among the 55 written responses included requests to: (a) make the trainings engaging (*“Make it hands on learning experience, and please make it as engaging as possible for the students.”*), (b) case based (*“active learning-- not slide sets!;” “Use-case driven training!”)*, and (c) based on real-world examples (*“Real-world examples are always helpful!;” “Most trainings that give real world examples of translation science in action are more impactful.”*).

## Discussion

Our findings add to the emerging methods for operationalizing TS principles to support evaluation of TS workforce development efforts. We found the seven TS principles coalesced into a smaller number of factors. However, the number and composition of these factors differed in our research focused on self-assessment of confidence in the TS principles versus content analysis of pilot grants [11]. We found TS principles organized into four factors related to Taking Risks for Impact, Multi-perspective Research, Collaboration Methods, and Efficiency and Productive Failure. In contrast, the prior study synthesized six TS principles (i.e., they did not include the principle focused on boundary-spanning partnerships) into three factors focused on Disruptive Innovation, Generalizability/Efficiency, and Team Science [11]. These differences may be due to variabilities in data collection methods (i.e., self-assessment versus content analysis), sample sizes, and/or because we included some new items in our research. Despite these differences, both studies revealed a common finding – the TS principles are inter-related and measures ought to take this into account.

Our findings revealed self-rated confidence related to the seven TS principles could be measured using a brief 14-item scale with good-to-excellent reliability. The four sub-scales within our brief scale also demonstrated good-to-excellent reliability. Having a brief scale may support adoption for evaluation due to decreased burden for data collection. Additional research is needed to evaluate how our brief scale performs with different populations and examine its sensitivity for capturing changes in confidence resulting from workforce development interventions.

Our findings also highlight opportunities to improve capacity among the clinical and translational science workforce related to the TS principles. Across all four factors, we found the mean level of confidence ranged from “slightly” to “moderately” confident among people affiliated with CTSAs. This aligns with our findings that most participants reported no formal training in TS.

### Ideas for TS workforce development

Our findings provide guidance for future TS workforce development. Participants reported TS trainings might be more desirable if they are virtual, multi-session, application-oriented, and real-world case based. TS trainings might benefit from lessons learned by other TS workforce development programs [15–17]. The two factors with the lowest levels of confidence among the participants were related to Efficiency and Productive Failure and Taking Risks for Impact. Next, we provide ideas for expanding capacity related to these two factors based on the items included in each factor.

TS workforce development activities focused on increasing researchers’ confidence to adopt more efficient processes and support productive failure are warranted to accelerate the pace of translational research. Accordingly, TS workforce development programs may help people navigate access to key resources designed to reduce time lags in research and get feedback as you go. Trainings may, for instance, build capacity to use shared administrative infrastructure to quickly bring teams together and execute grants and contracts or shared resources (e.g., instrumentation, sample storage, community engagement) to rapidly deploy resources as opportunities emerge [18]. TS workforce development efforts should support research staff and investigators as they navigate access to these resources. TS workforce development might also focus on enhancing capacity to use milestone-based planning, including prespecified “go/no-go” decision making, to support data-driven adaptation when the best laid plans are not working [19].

TS workforce development activities focused on increasing confidence among researchers to take risks for impactful research are also warranted because “risky research generates the groundbreaking advances that expand knowledge most rapidly” [20]. Risky research is needed to address persistent TS challenges leading to the “avalanche of successful fundamental discovery” not leading to “expected therapeutic windfall” [2]. This may include TS workforce development efforts to increase confidence among research staff and investigators to conduct high risk/high reward research focused on rate limiting factors influencing the pace at which evidence-based treatments, diagnostics, or interventions are adopted in clinical and public health settings. This type of TS workforce development may target individual-level capacity building by improving skills to conduct integrated team science, meaningfully engage historically excluded communities in research, enhance research program management to support teams along the “multistep, multidomain, multidiscipline recursive web” of TS, and link to resources supporting dissemination of findings that foster real-world application in settings with the most unmet needs [2].

Corollary institutional infrastructure is needed to encourage high risk research among the TS workforce while attending to possibilities of either transformation (in the best case) or failure (in the worst case). Institutionalized reward structures (e.g., tenure, honors) within the scientific ecosystem (e.g., academia, funding, societies) tend to reward the compilation of research outputs (e.g., publications, grants) [16]. These outputs may not reflect the hours worked or risks taken within scientific endeavors that failed in so far as they did not result in a legitimized output [20]. This can lead to a system in which only those with prior success accelerate in the research process, even if those prior successes only incrementally expanded the field of TS. Accordingly, some have called for TS to shift attention away from “proving” and more to “improving” through quality improvement models (i.e., Plan-Do-Study-Act, Root Cause Analysis) to encourage risk taking for impactful research [21]. This will require shifts in institutionalized reward systems to flip the script that productive failure is valued.

Taken together, these highlight tensions between measurement and training related to the TS principles. The brief scale we developed to measure confidence in the TS principles might support better understanding of gaps in capacity to tailor training. Workforce development approaches designed to boost capacity related the TS principles ought to account for the range of individual, institutional, and systematic forces shaping TS confidence. Trainings that fail to address key barriers to accelerating translational research, such as reward systems, might not translate into improvements in the goal of getting better health solutions to the right people and places at the right time.

### Limitations

Our research has some limitations. The sample was limited to researchers, staff, and partners affiliated with three CTSA hubs. While this expands representation, it does not include perspectives from all potential sites. Findings are based on self-report, which may introduce bias.

### Conclusion

In summary, our findings provide a brief tool for assessing confidence related TS principles. This may serve as a resource for evaluating future TS workforce development interventions. Our findings amplify clear need and strong interest for enhanced TS workforce development, emphasizing training approaches that are virtual, application-oriented, and based on real-world cases. Continued research will be critical to refine workforce development initiatives and robustly evaluate their effectiveness in cultivating confidence across the TS principles and the extent to which these changes result in more TS benefits [22].

## Previously presented

This research has not been previously presented. It is accepted for presentation at the Translational Science Conference, Milwaukee, WI, April 2026.

## Table 1. Long description

The table presents the characteristics of study participants, including race, ethnicity, gender, age group, professional affiliation, role, amount of time spent conducting health-related research, translational research focus, and prior formal training in translational science. The table has 15 rows and 3 columns. The columns are labeled Race, N, and Percent. The rows are labeled with different races and ethnicities, gender, age groups, professional affiliations, roles, amount of time spent conducting health-related research, translational research focus, and prior formal training in translational science. The values in the table represent the number and percentage of participants in each category. For example, 107 participants are White, which is 68 percent of the total participants. 119 participants are women, which is 75 percent of the total participants. 78 participants are affiliated with healthcare, pharmaceutical, or device sectors, which is 49 percent of the total participants. 55 participants are research staff, which is 35 percent of the total participants. 39 participants spend minimal time conducting health-related research, which is 25 percent of the total participants. 79 participants focus on pre-clinical research, which is 50 percent of the total participants. 60 participants have minimal prior formal training in translational science, which is 38 percent of the total participants.

Navigate back to Table 1..

## Figure 2. Long description

The plot depicts confidence in translational science principles organized by participant age, showing self-reported confidence levels across four factors among different age groups. The four factors are Taking Risks for Impact, Multi-perspective Research, Collaborative Methods, and Efficiency and Productive Failure. The age groups are 18-29 years, 30-45 years, 46-60 years, and more than 60 years, represented by purple, turquoise, green, and red colors respectively. The x-axis represents the average confidence score ranging from 1 to 5, and the y-axis lists the four factors. Each age group’s confidence rating is shown with a dot and a horizontal line indicating the 95% confidence interval. The plot shows that there are no significant differences by age group across the four factors. The data points for each age group and factor are as follows: Factor 1: Taking Risks for Impact: 18-29 years (3.06), 30-45 years (2.65), 46-60 years (2.88), More than 60 years (2.73). Factor 2: Multi-perspective Research: 18-29 years (3.42), 30-45 years (3.46), 46-60 years (3.45), More than 60 years (3.44). Factor 3: Collaborative Methods: 18-29 years (3.46), 30-45 years (3.14), 46-60 years (3.36), More than 60 years (3.3). Factor 4: Efficiency and Productive Failure: 18-29 years (2.86), 30-45 years (2.41), 46-60 years (2.66), More than 60 years (2.54).

Navigate back to Figure 2..
